# Maternal Vitamin D Status and Delivery by Cesarean

**DOI:** 10.3390/nu4040319

**Published:** 2012-04-20

**Authors:** Theresa O. Scholl, Xinhua Chen, Peter Stein

**Affiliations:** 1 Two Medical Center Drive, Department of Obstetrics and Gynecology, SOM, University of Medicine and Dentistry of New Jersey, Stratford, NJ 08084, USA; Email: chenx1@umdnj.edu; 2 Two Medical Center Drive, Department of Surgery, SOM, University of Medicine and Dentistry of New Jersey, Stratford, NJ 08084, USA; Email: tpstein@umdnj.edu

**Keywords:** pregnancy, 25 hydroxyvitamin D, vitamin D deficiency, vitamin D insufficiency, dietary vitamin D intake, dietary calcium intake, parathyroid hormone, cesarean delivery, prolonged labor

## Abstract

We examined the association of vitamin D deficiency to risk of cesarean delivery using prospective data in a cohort of 1153 low income and minority gravidae. Circulating maternal 25-hydroxyvitamin D and intact parathyroid hormone were measured at entry to care 13.73 ± 5.6 weeks (mean ± SD). Intake of vitamin D and calcium was assessed at three time points during pregnancy. Using recent Institute of Medicine guidelines, 10.8% of the gravidae were at risk of vitamin D deficiency, and 23.8% at risk of insufficiency. Maternal 25-hydroxyvitamin D was related positively to vitamin D and calcium intakes and negatively to circulating concentrations of parathyroid hormone. Risk for cesarean delivery was increased significantly for vitamin D deficient women; there was no increased risk for gravidae at risk of insufficiency. When specific indications were examined, vitamin D deficiency was linked to a 2-fold increased risk of cesarean for prolonged labor. Results were the similar when prior guidelines for vitamin D deficiency (25(OH)D < 37.5nmol/L) and insufficiency (37.5–80 nmol/L) were utilized.

## 1. Introduction

Delivery by cesarean is a common operative procedure experienced by reproductive age women [1]. A Cesarean may be performed for reasons related to the mother or to the fetus including prolonged labor (dystocia), fetal distress, fetal malpresentation or a prior cesarean delivery [2]. Factors which increase risk include older maternal age, obesity, parity and ethnicity [3,4] along with a more recently defined factor—maternal nutrition [5,6,7].

Recent research in the United States found low circulating 25-hydroxyvitamin D (25(OH)D), the primary indicator of vitamin D status, among women who were either pregnant or in their reproductive years [8,9,10]. Vitamin D is present in food either naturally or by fortification and included in nutritional supplements; the majority is synthesized photochemically by the skin from ultraviolet B radiation [11]. One way by which poor maternal vitamin D status might increase risk of cesarean delivery is by reducing strength of the pelvic musculature and the mother’s ability to push and deliver vaginally. However, two observational studies came to different conclusions; one reported an increased risk of cesarean for women with concentrations below 37.5 nmol/L [5] and the other no association with maternal 25(OH)D [6].

The extent to which maternal vitamin D influences the course and outcome of human pregnancy remains to be more completely studied. We used an HPLC method to assay circulating 25(OH)D to assess the influence of maternal vitamin D at entry to care on risk of cesarean delivery in a cohort of young, low income minority gravidae from Camden, New Jersey.

## 2. Methods

The Camden Study examines the effects of maternal nutrition and metabolism in generally healthy pregnant women from one of the poorest cities in the United States [7,12]. Participants include teenage and mature women enrolling for prenatal care in Camden clinics. Gravidae with serious non-obstetric problems (e.g., lupus, chronic hypertension, diabetes mellitus Type 1 or Type 2, and seizure disorders, malignancies, drug or alcohol abuse as verified from the medical record) are not eligible. Approximately 80% of eligible gravidae agreed to participate. The Institutional Review Board of the University of Medicine and Dentistry of New Jersey approved the study. In this analysis, we focused on 25(OH) D in gravidae enrolled and delivered from 2001 to 2007. 

Socioeconomic, demographic, lifestyle, and dietary data were obtained by interview at entry to prenatal care, and updated at weeks’ 20 and 28 gestation. A 24-h recall of the previous day’s diet was obtained on the same schedule, processed with databases from the Campbell Institute of Research and Technology (Campbell Soup Company) in Camden. The database generates data for more than 70 nutrients using the United States Department of Agriculture Nutrient Database for Standard Reference [13] and the Continuing Survey of Food Intakes by Individuals [14] as well as data from the scientific literature. Intakes have been validated by computing measures of reliability and by assay of some circulating biomarkers [15,16,17,18,19]. The nutrient values of vitamin D and calcium from diet and from diet plus supplements were averaged across the pregnancy and the mean used in this analysis; entry data were included for comparison.

BMI was computed (kg/height (m^2^)) from recalled pregravid weight and height measured with a stadiometer at entry to care. Maternal ethnicity (African American, Hispanic and white) was self reported. Information on presence or absence of cesarean delivery, whether this was a primary (no prior cesarean) cesarean along with the indication for the cesarean were abstracted from the delivery record, and delivery logbooks. A total of 56/290 (19.3%) had an elective cesarean, the remainder were unplanned. 

Circulating 25(OH)D was measured as 25 hydroxyvitamin D_3_ (25(OH)D_3_) and 25 hydroxyvitamin D_2_(25(OH)D_2_) by HPLC in serum [20] using a kit marketed by Chromosorb (Germany). Briefly, to 0.5 mL of serum are added 350 µL of methanol–2-propanol (80:20 by volume) and the 25(OH)D extracted by mixing three times with 2 mL of hexane. The phases were separated by centrifugation, and the upper organic phases combined and dried under nitrogen. The residue was then dissolved in 100 µL of mobile phase. Calibration curves were constructed using four concentrations of 25(OH)D (15–120 nmol/L) and human serum albumin (50 g/L). For the chromatography we used a Waters Millenium HPLC (Waters Inc., Milford MA) fitted with a LiChrospher 60 RP select B column (4 × 250 mm; 5 µm bead size; EMD, Bridgewater, NJ) maintained at 40 °C. The separation was achieved using 760 mL/L methanol in water as the mobile phase with a flow rate of 1 mL/min and detection at 265 nm. The injected volume was 50 µL. The 25(OH)D_3_ and 25(OH)D_2_ peaks are completely resolved with retention times of 20.8–21.1 min and 23.1 min, respectively. The within-assay and between assay CVs were <8%.

Serum intact parathyroid hormone (PTH) was measured by immuno-radiometric assay (IRMA) (DSL Diagnostic Systems Laboratories, Inc., Webster, Texas). The two-site IRMA is a non-competitive assay using two antibodies directed to non-overlapping *N*-terminal and *C*-terminal PTH fragments respectively. When these two antibodies are paired in a two-site IRMA, only intact PTH is measured. The overall intra and inter-assay coefficients of variation were <5%. Maternal serum obtained at entry was stored at −70 °C and used for the assays. Available data show that 25(OH)D and PTH are stable and reproducible for several years when stored at −70 °C [21,22]. 

## 3. Data Analysis

The association of maternal characteristics, PTH and intake of vitamin D and calcium during the pregnancy with circulation 25(OH)D was assessed for trend (Chi Square, ANOVA). We used ordinary least squares regression to determine the relation of PTH to 25(OH)D examining linear, quadratic and quartic terms to fit the line.

Concentrations of 25(OH)D_2_ were very low so that vitamin D status (25(OH)D) was based mainly on 25(OH)D_3_. Following recent Institute of Medicine (IOM) guidelines [11] we utilized 25(OH)D at <30.0 nmol/L (<12 μg/L) to indicate risk of vitamin D deficiency; concentrations from 30.0–49.9 nmol/L (12–20 μg/L) to indicate risk of insufficiency. Serum concentrations between 50 and 125 nmol/L, deemed vitamin D sufficient [11], were used as the reference group. In addition, we also compared these results to those obtained using prior guidelines [23]: <37.5 nmol/L, 37.5–80.0 nmol/L and >80 nmol/L.

Multiple logistic regression was used to fit separate models for cesarean delivery (total and primary cesarean). Gravidae were compared to those whose 25(OH)D was sufficient. Polytomous (multinomial) logistic regression is an extension of traditional logistic regression which models multiple level outcomes so that odds ratios and adjusted odds ratios for more than one outcome are estimated in a single model [24]. Since we hypothesized that poor maternal vitamin D status was associated with poor muscle tone and ability to push we estimated the relation of 25(OH)D to two indications for cesarean: prolonged labor (dystocia) and fetal distress. We also included all other indications for cesarean in the same model. All models were adjusted for potential confounding variables associated with adverse outcomes in Camden or from the published literature including age, parity, ethnicity, smoking, pregravid BMI, gestation at entry and season at entry. Data were analyzed with SAS version 9.0 (SAS Institute, Cary, NC). 

## 4. Results

Table 1 gives entry data 13.73 ± 5.6 (SD) completed weeks gestation] for the cohort of 1153 low income and minority women categorized by concentrations of 25(OH)D suggestive of deficiency and insufficiency. Concentrations of 25(OH)D_2_ were very low and fewer than one quarter of the women had detectable levels (greater than zero) thus maternal 25(OH)D consisted mainly of 25(OH)D_3_.

By recent Institute of Medicine criteria, 10.8% (*N* = 125) of the cohort had concentrations of 25(OH)D placing them at risk of vitamin D deficiency, and an additional 23.85% (*N* = 275) had concentrations suggesting insufficiency. There were significant linear trends for parous women, for women of African American or Hispanic ethnicity, for those with BMIs indicating overweight (25–29.9) or obesity (≥30), and for women entering care during the winter (January-March) to be at higher risk of deficiency and insufficiency (Table 1). On the other hand, women with levels >125 nmol/L were more likely to be nulliparous and white, to have a low BMI (<25) and to enter during the summer (July–September) (Table 1).

**Table 1:** Linear trends for maternal characteristics and intact parathyroid hormone according to categorized 25(OH)D.

Characteristics	*N*	25(OH)D Concentration				*p* for Trend
		<30.0 nmol/L (*N* = 125)	30–49.9 nmol/L (*N* = 275)	50–125 nmol/L (*N* = 683)	>125 nmol/L (*N* = 70)	
25(OH)D (nmol/L) Mean, SEM	1153	22.6 (0.49)	40.7(0.33)	77.6(0.75)	142.1(1.81)	<0.001
25(OH)D_3_ (nmol/L) Mean, SEM	1153	22.2 (1.49)	39.5(1.00)	75.5(0.64)	139.7(2.0)	<0.001
25(OH)D_2_ (nmol/L) Mean, SEM	1153	0.44 (0.52)	1.16 (0.35)	2.10 (0.22)	2.34 (0.70)	0.007
% Detectable		12.0	22.9	25.9	28.6	<0.005
Intact Parathyroid Hormone (pmol/L)	1141	5.6 (0.21)	4.5 (0.14)	3.6 (0.09)	3.4 (0.27)	<0.001
Gestation at Entry (weeks) Mean, SEM	1153	13.0 (0.50)	13.5 (0.34)	13.8 (0.21)	15.5 (0.67)	0.014
Age (years) Mean, SEM	1153	23.0 (0.50)	22.7 (0.33)	22.9 (0.21)	22.2 (0.66)	0.67
Parity (%)						
Nulliparous	435	8.5	18.2	65.3	8.1	<0.001
Parous	718	12.3	27.3	55.6	4.9	
BMI (kg/m^2^) Mean, SEM	1153	27.5 (0.57)	27.5 (0.38)	25.6 (0.24)	22.9 (0.77)	<0.001
BMI (%)						
<25	619	9.5	19.2	62.5	8.7	<0.001
25–29.9	237	13.1	25.7	58.2	2.9	
≥30	297	11.8	32.0	53.2	3.0	
Smoking (%)						
Yes	235	11.5	22.1	58.7	7.7	0.65
No	918	10.7	24.3	59.4	5.7	
Ethnicity (%)						
African-American	399	18.3	33.3	46.9	1.5	<0.001
Hispanic	593	8.4	21.6	62.7	7.3	
White	161	1.2	8.7	77.0	13.0	
Season at Entry (%)						
Winter	306	19.0	27.1	49.7	4.3	<0.001
Spring	324	11.7	25.6	56.8	5.9	
Summer	257	2.7	14.8	74.7	7.8	
Fall	266	8.3	26.7	58.3	6.8	
Insurance Source (%)						
Medicaid	1137	11.0	23.7	59.1	6.2	0.59
Other	14	10.0	78.6	21.4	0	

There was a significant trend for PTH concentrations to decrease according to the categories of 25 (OH) D shown in Table 1; a comparison of the PTH at 50–125 nmol/L 25(OH)D to those >125 nmol/L suggested no difference between the two (*p* = 0.45). Circulating 25(OH)D and PTH were negatively correlated (*r* = −0.26) so that PTH concentrations fell as circulating 25(OH)D increased.

**Figure 1:**
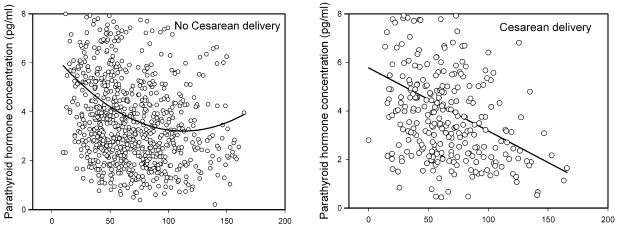
Serum 25-hydroxyvitamin D and parathyroid hormone in women without (left panel) and with (right panel) cesarean delivery. Data are unadjusted.

Figure 1 graphically plots unadjusted serum 25(OH)D and PTH for gravidae with and without a cesarean delivery. A straight line gave the best fit for women delivered by cesarean (−0.019 ± 0.004 pmol/L (b,SE), *p*< 0.001). A quadratic function provided the best fit for women without a cesarean [(−0.047 ± 0.009 pmol/L) + (0.0002 ± 0.000055 pmol/L^2^ (b,SE))] *p* < 0.001 for each. When linear models were fit for each group, adjusted (age, parity, BMI, ethnicity, smoking, season and gestation at entry) and compared the slopes differed significantly (*p* = 0.035); thus gravidae delivered by cesarean had more PTH per unit of 25(OH)D than women without a cesarean.

Vitamin D and calcium in the maternal diet or diet plus supplements increased as 25(OH)D rose. Intakes of vitamin D and calcium (diet or diet plus supplements) were usually lower when circulating 25(OH)D fell to <30 nmol/L but increased regularly between 30 and 49.9 nmol/L and at or above 50 nmol/L. This was true regardless of whether the overall mean from the pregnancy was utilized or data from the entry visit alone (Table 2). The result was the similar when 25(OH)D concentrations <37.5 and between 37.5 and 80 were compared to concentrations exceeding 80 nmol/L (data not shown). 

**Table 2:** Energy adjusted intake of vitamin D and calcium from diet and diet plus supplements by 25(OH)D concentration.

Intake(Mean, SEM)	25(OH)D Concentration				*p* for Trend
	<30 nmol/L	30–49.9 nmol/L	50–125 nmol/L	>125 nmol/L	
	(*N* = 125)	(*N* = 275)	(*N* = 683)	(*N* = 70)	
Vitamin D (diet μg/day)					
Mean ^1^	4.0 (0.29)	4.1 (0.19)	4.9 (0.12)	5.2 (0.38)	<0.001
Entry	3.6 (0.38)	3.5 (0.27)	4.6 (0.16)	5.2 (0.53)	<0.001
Calcium (diet mg/day)					
Mean ^1^	876 (30.5)	868 (20.8)	943 (13.0)	985 (40.9)	<0.001
Entry	821 (44.3)	809 (31.5)	917 (19.3)	992 (63.1)	<0.001
Vitamin D (diet + supplements μg/day)					
Mean ^1^	9.1 (0.34)	9.5 (0.23)	10.6 (0.15)	11.2 (0.46)	<0.001
Entry	8.6 (0.43)	9.1 (0.31)	10.6 (0.19)	11.1 (0.62)	<0.001
Calcium (diet + supplements mg/day)					
Mean ^1^	1021 (30.7)	1024 (21.0)	1110 (13.1)	1156 (41.2)	<0.001
Entry	963 (44.3)	969 (31.5)	1089 (19.3)	1160 (63.1)	<0.001

^1^ Mean of 3 intakes (Entry, 20, 28 Weeks), energy adjusted.

A total of 290 gravidae from the cohort (25.2%) were delivered by cesarean of which 173 (15.0%) had primary cesareans. Using concentrations of 25(OH)D suggestive of sufficiency as the reference and controlling for potential confounding variables apart from BMI, the increase in risk for cesarean and primary caesarean were significant for gravidae at risk of deficiency (25(OH)D < 30 nmol/L). After control for BMI the increase was significant and less than 2-fold (66%) for total caesarean; primary cesarean was similarly increased (68%) but 95% confidence intervals now included unity (*p* = 0.054). Gravidae at risk for insufficiency (30–49.9 nmol/L) did not have an increased risk for total or primary cesarean (Table 3). 

**Table 3:** Maternal 25(OH) D and Cesarean Delivery (primary, total).

**Total Cesarean Deliveries**						
**25(OH)D Concentration**	***N***	**%**	**AOR ^1^**	**95% CI**	**AOR ^2^**	**95% CI**
<30 nmol/L	125	35.2	1.70	1.12, 2.58	1.66	1.09, 2.52
30–49.9 nmol/L	275	22.2	0.89	0.63, 1.25	0.83	0.59, 1.17
50–125.0 nmol/L	683	24.9	Reference	-	Reference	-
>125 nmol/L	70	21.4	0.59	0.17, 2.08	0.90	0.49, 1.66
**Primary Cesarean Delivery ^3^**						
**25(OH)D Concentration**	***N***	**%**	**AOR ^1^**	**95% CI**	**AOR ^2^**	**95% CI**
<30 nmol/L	105	22.9	1.79	1.07, 3.01	1.68	0.99, 2.84
30–49.9 nmol/L	247	19.1	0.89	0.58, 1.37	0.80	0.52, 1.24
50–125.0 nmol/L	618	17.0	Reference	-	Reference	-
>125 nmol/L	66	16.7	0.94	0.47, 1.87	1.03	0.51, 2.06

^1^ Adjusted for age, parity, ethnicity, smoking, gestation at entry and season at entry; ^2^ Adjusted for age, parity, ethnicity, smoking, gestation at entry, season at entry and BMI; ^3^ Excludes gravidae with a second cesarean section.

We found similar but slightly stronger results comparing concentrations of 25(OH)D < 37.5 nmol/L to those in excess of 80 nmol/L. After control for potential confounding variables including BMI, there was approximately a 2-fold increase in risk for a primary cesarean (Adjusted Odds Ratio (AOR) = 1.99, 95% Confidence Interval (CI): 1.20, 3.30) and a less than 2-fold increase (AOR = 1.74, 95% CI: 1.13, 2.67) for total cesarean delivery with 25(OH)D concentrations below 37.5 nmol/L; risk was not increased between 37.5 and 80 nmol/L (AOR = 1.25, 95% CI: 0.81, 1.24 for primary cesarean and AOR = 1.14, 95% CI: 0.80, 1.59 for total cesarean). 

A total of 7.7% (*N* = 89) of the cohort were delivered by cesarean for a prolonged labor and another 4.9% (*N* = 56) for fetal distress. These two causes together accounted for more than half (52.8%) of all deliveries by cesarean. Other causes included elective cesarean (4.9%), fetal malpresentation (2.5%), failed induction (1%) preeclampsia (0.5%), placental abruption (0.9%) and others (3.3%). Risk of cesarean for prolonged labor was increased 2-fold for gravidae with 25(OH)D < 30 nmol/L. No increase in risk for fetal distress as a cause of cesarean or for causes other than prolonged labor or fetal distress was noted among gravidae with 25(OH)D < 30 nmol/L or between 30 and 49.9 nmol/L (Table 4). When data were restricted to women with a primary cesarean and 25(OH)D was <30 nmol/L there was a 2-fold increase in cesarean for prolonged labor (AOR = 2.12, 95% CI 1.06, 4.32 after control for potential confounders) but no increase in fetal distress or other causes of cesarean delivery; no increase in risk was found when levels fell between 30 and 49.9 nmol/L. A similar result was noted for prolonged labor (AOR = 2.24, 95% CI: 1.17, 3.98) when gravidae with concentrations falling below 37.5 nmol/L 25(OH)D were compared to those >80 nmol/L after control for potential confounding variables; no increased risk was noted when 25(OH)D was between 37.5 and 80 nmol/L (AOR = 1.18, 95% CI: 0.68, 2.06).

**Table 4:** Maternal 25(OH) D and total cesarean by cause.

25(OH)D Concentration	*N*	Prolonged Labor			Fetal Distress			Other Causes		
		%	AOR ^1^	95% CI	%	AOR ^1^	95% CI	%	AOR ^1^	95% CI
<30 nmol/L	125	12.0	2.08	1.09, 3.98	5.6	1.54	0.64, 3.70	17.6	1.38	0.79, 2.38
30–49.9 nmol/L	275	8.0	1.14	0.66, 1.97	5.1	1.09	0.56, 2.11	9.1	0.56	0.35, 0.92
50–125.0 nmol/L	683	7.0	Reference	-	4.5	Reference	-	13.3	Reference	-
>125 nmol/L	70	5.7	0.81	0.28, 2.35	5.7	1.21	0.41, 3.60	10.0	0.82	0.36, 1.93

^1^ Adjusted for age, parity, ethnicity, smoking, BMI, gestation at entry and season at entry.

## 5. Discussion

In our prospective study of low income and minority Camden gravidae, the proportion at risk of vitamin D deficiency (<30.0 nmol/L) was 10.8%; with another 23.8% at risk of insufficiency, neither differs markedly from prevalence data reported by the Centers for Disease Control using Institute of Medicine guidelines [25]. The indications for cesarean in this study, including the leading indication (prolonged labor) were the same as for an international comparison which included the United States [2]. We found that risk of vitamin D deficiency at entry to care linked to an increased risk of cesarean delivery as well as to a better than two fold increase in risk for a specific indication for cesarean: prolonged labor. This was true regardless of whether we used a lower (<30 nmol/L) or a higher (<37.5 nmol/L) concentration of 25(OH)D to index vitamin D deficiency. There was no increase in risk of cesarean for women with vitamin D insufficiency however defined. There was no increased risk for women with levels of 25(OH)D exceeding 125 nmol/L. Higher circulating levels of 25 (OH)D have been related to increased mortality and risk of certain cancers but are not thought to be of concern during pregnancy [11]. Recent Endocrine Society guidelines [26] recommend a range of 25(OH)D between 100–150 nmol/L for adults including pregnant women with only extraordinary levels (e.g., 25(OH)D > 375 nmol/L) warranting concern about toxicity.

These data are consistent with a study by Merewood [5] where women with a primary cesarean section had lower concentrations of 25(OH)D measured within 72 h of delivery than controls who delivered vaginally. While they did not examine the indications for cesarean, a small case-control study of Pakistani women looked at a specific cause (cesarean for obstructed labor from cephalopelvic disproportion) but reported no association with 25(OH)D at delivery [6]. 

Vitamin D receptors are present in skeletal muscle [27]. Vitamin D deficiency and insufficiency are related to muscle mass and strength in younger women [28,29]. Circulating levels of 25(OH)D correlated positively with jumping mechanography, a measure of muscle force and power, in young women from Manchester, England; the mean 25(OH)D concentration of participants was 28.9 nmol/L [28]. Chinese adolescents who were vitamin D deficient (25–50 nmol/L) or severely deficient (<25 nmol/L) had reduced handgrip muscle strength when compared to those with higher levels of 25(OH)D [29]. In a cross sectional study of US adults, lower 25(OH)D was associated with less skeletal muscle mass in younger women (range 21–64 years) but not reduced grip strength [30]. While there are few studies of vitamin D and muscle strength in reproductive age women, recent research using data from NHANES 2005–2006 showed a decreased risk of pelvic floor disorders for women with concentrations of 25(OH)D exceeding 75 nmol/L. For women age 20 and older risk was reduced by 6% for each 5 unit increase in 25(OH)D and by 8.6% for women aged 50 and over [31]. During pregnancy strengthening muscles of the pelvic floor enhances muscle control and flexibility, prevents urinary incontinence during and after delivery [32] and smoothes the progress of labor [33]. In one study, gravidae randomly assigned to an exercise regimen to strengthen their pelvic floor musculature had lower rates (22%) of a prolonged second stage of labor compared to controls (37%) [33]. Thus it is plausible that one way by which poor maternal vitamin D status increases risk of cesarean delivery is by reducing pelvic muscle strength and control leading a reduced ability to push and to a longer and more difficult labor. 

Since our study was prospective, *i.e.*, examined the influence of 25(OH)D months before delivery, supplementation of women at risk of vitamin D deficiency from early pregnancy onward might reduce the rate of cesarean delivery. In a recent randomized controlled trial, 25.3% of women assigned to receive 400 IU vitamin D/day, 20.6% assigned to 2000 IU/day and 14.3% assigned to 4000 IU/day delivered by cesarean. These results were not statistically significant owing, in part, to small sample size (111–122/group) [34]. 

Most of the 25(OH)D measured in our cohort was 25(OH)D_3_ which is also synthesized by the skin after exposure to ultraviolet B radiation from the sun whereas 25(OH)D_2_ is not [11,23]. Vitamin D is present in few foods naturally (fatty fish, fish liver oils), used to fortify others (milk, breakfast cereals, orange juice), and included in nutritional supplements (prenatal multivitamins). Vitamins D_2_ and D_3_ are both used in nutritional supplements and for food fortification [11]. We found that calcium and vitamin D from diet or diet plus supplements was positively related to circulating 25(OH)D. Mean dietary intakes of vitamin D and calcium averaged over the pregnancy were similar to NHANES III data for reproductive age women [35]. Mean intake from diet plus supplements approximated the pregnancy EAR for calcium at all concentrations of 25(OH)D and the pregnancy EAR for vitamin D when circulating 25(OH)D was 50 nmol/L or better [11]. The Endocrine Society’s guidelines call for a higher vitamin D intake in at risk pregnant women with a range of 15–25 μg/day in women age 18 and younger and 35.5–50 μg/day in women 19 and older [26].

Parathyroid hormone and 25(OH)D are inversely related; an increased concentration of PTH is a functional indicator of vitamin D deficiency and insufficiency. Consistent with others we found that as circulating concentrations of 25(OH)D decreased, PTH rose [11,23]. In our study PTH was increased at concentrations suggestive of deficiency and insufficiency, declined as 25(OH)D increased and was no different when concentrations consistent with vitamin D sufficiency and those exceeding 125 nmol/L were compared. The shape of the curve describing the relation between PTH and 25(OH)D was different for women delivered vaginally (quadratic) or by cesarean (linear). When both groups were compared using the same model, the slopes were significantly different and suggested more PTH per unit of 25(OH)D in women delivered by cesarean. 

Finally, we confirmed vitamin D variation with maternal BMI, by season of the year and according to maternal ethnicity and found that poorer vitamin D status occurs with overweight and obesity [36,37] during the winter months and with increasing intensity of skin pigmentation [8]. While we relied on the medical record to identify women with prolonged labor, future studies would benefit from use of a more uniform definition. Results are also limited by use of self declared race/ethnicity as a proxy for skin pigmentation and season of year as a surrogate for sun exposure. 

## 6. Conclusions

While there is no single reason for the continued rise in cesarean delivery in the United States, our data suggest that decreasing the number of women at risk of vitamin D deficiency might have important ramifications for women, their pregnancies and the cost of their care. Results stemming from observational studies, even when they are prospective, can be endlessly debated, if for example, they are a consequence of uncontrolled confounding or of differences in medical judgment. But the question at hand about vitamin D and cesarean delivery is one that is best answered by an experiment, a randomized controlled trial of vitamin D supplementation in a population with poor vitamin D status where the doses are high enough to move women into ranges of 25(OH)D associated with decreased risk [5,38].
